# Spicy Food Ingredient from Red Habanero By-Product Obtained by Ultrasound-Assisted Extraction

**DOI:** 10.3390/foods14081407

**Published:** 2025-04-18

**Authors:** António Toscano, Andreia F. R. Silva, Maria P. Ramos, Norton Komora, Filipa V. M. Silva, Patrícia Fradinho

**Affiliations:** 1 Instituto Superior de Agronomia, Universidade de Lisboa, Tapada da Ajuda, 1349-017 Lisboa, Portugal; antonio.graca.4@gmail.com; 2 Colab4Food—Collaborative Laboratory for Innovation in the Agri-Food Sector, Rua dos Lagidos, Vairão, 4485-655 Vila do Conde, Portugal; andreia.silva@colab4food.com; 3 Casa Mendes Gonçalves, Zona Industrial, Lote 6, 2150-268 Golegã, Portugal; maria.ramos@casamg.pt (M.P.R.); norton.komora@casamg.pt (N.K.); 4 LEAF—Linking Landscape, Environment, Agriculture and Food Research Center, Instituto Superior de Agronomia, Universidade de Lisboa, Tapada da Ajuda, 1349-017 Lisboa, Portugal; 5 Associate Laboratory TERRA, Instituto Superior de Agronomia, Universidade de Lisboa, Tapada da Ajuda, 1349-017 Lisboa, Portugal

**Keywords:** *Capsicum chinense*, waste, oleoresin, food colorants, circular economy, sustainability

## Abstract

The production of spicy sauces from chili peppers (*Capsicum* spp.) generates 5–30% of spicy by-product which is rich in valuable compounds (e.g., capsaicinoids, carotenoids, phenolics, etc.) and can serve as a source of *Capsicum* oleoresins, providing spice and color ingredients for food products. This study primarily focused on the optimization of *Capsicum* oleoresin extraction from Red Habanero chili pepper (*Capsicum chinense* Jacq.) by-product using ultrasound-assisted extraction (UAE). A second focus was the comparison between UAE and reflux-assisted extraction (RAE). Response Surface Methodology (RSM) was employed to optimize the extraction time (3 to 17 min) and acoustic power density (APD, 0.30 to 1.00 W/mL). The optimal UAE conditions (8 min, 0.87 W/mL) showed a higher extraction yield (26%) and high quality oleoresin extracts rich in bioactives (capsaicinoids: 7 mg/g; phenolics: 4 mg GAE/g) with antioxidant activity (FRAP: 139 µmol FeSO_4_ eq/g; DPPH: 33 µmol TEAC/g). Optimum UAE extracts proved more colored, energy-efficient (95% less consumption), equally spicy (466,000 SHU) and had higher antioxidant activity than RAE. These results demonstrated UAE as a sustainable method for producing high value spicy additives from chili pepper by-product, turning them into products with enhanced bioactivity, favoring a circular economy in the agri-food industry.

## 1. Introduction

In the hot sauce industry, about 5–30% of spicy by-product is generated [1]. Chili pepper (*Capsicum* spp.) by-product is currently used as organic fertilizer, although this practice has been questioned by Costa et al. [2] and Zupančič et al. [3], who raised concerns whether this practice poses environmental risks due to the by-product’s rich composition of aromatic chemical compounds, namely capsaicinoids, polyphenols, flavonoids, and sauce formulation ingredients such as salt, vinegar, garlic, and other seasonings. This waste, if not properly treated and managed, may pose ethical and environmental problems, either for containing nutritional value in the form of high-quality dietary fiber, protein, and edible oils [4,5], or by boosting environmentally impactful gas emissions or potentially contaminating soils and water bodies, ultimately disrupting the ecosystem’s balance [6].

From an industrial perspective, chili pepper processing by-product can still hold value, as it still retains a considerable amount of spicy compounds (secondary chili pepper exclusive metabolites named capsaicinoids) with concentrations ranging from 102 mg to 450 mg per gram [7]. In fact, the presence of large amounts of capsaicinoids in chili pepper by-product potentially comes from fruit placenta and seeds (responsible for this compound’s synthesis and storage), which are removed from the sauce production line as those parts of the fruit are considered non-edible [8]. Capsaicinoids alone can contribute to about 80% of the bioactivity and functional properties of the original fruit [9], namely its high antioxidant and antimicrobial properties, alongside with positive food functional aspects such as its usage as a stabilizer and preservative [10,11]. Furthermore, the spicy and “hot” sensations associated with these compounds’ consumption are recognized for nutraceutical and pharmacological beneficial effects such as metabolism acceleration [12] and fat-oxidation [13], altogether contributing to reducing obesity [14] and therefore increasing consumers interest and demand.

Parallel to capsaicinoids, these leftover parts are also rich in natural vegetable pigments such as capsanthin, capsorubin, beta-carotene, zeaxanthin, and violaxanthin, which are well recognized in literature by their red/orange natural coloration [15], pharmacological effects [16], and for being precursors to vitamin A [17]. The most relevant pigments are capsanthin and capsorubin, whose synthesis and presence is also exclusive to the fruits of the *Capsicum* genus, largely found in chili pepper skin and peels, contributing to the characteristic intense dark red coloration of peppers and their derived products [18]. These compounds can serve as protectors of human organs’ DNA such as skin [19]. Furthermore, there is strong evidence that these pigments can hinder cancer formation [20]. Carotenoids are still largely present in chili pepper sauce processing by-product, as demonstrated by Vulić et al. [21].

Regarding phenolic compounds, which are vegetable secondary metabolites and precursors to capsaicinoids when fused with free fatty-acids, their chemical aromatic ring structure and derived functional properties are considered relevant, especially for their bioactive and nutraceutical effects [22]. These compounds are recognized in the literature as possessing potent antioxidant and antimicrobial properties, as recently studied by Lekmine et al. [23]. The most common phenolic compounds found in chili pepper fruits are vanillic, caffeic acid, ferulic acid, and p-coumaric (phenolic acids) and quercetin, luteolin, and kaempferol (flavonoids). As demonstrated by Cortés-Ferré et al. [24], who performed an enzyme assisted extraction on Red Habanero (*Capsicum chinense* Jacq.) chili pepper by-product, the waste parts still contain high concentrations of vanillic acid (7.97–12.66 mg/g) and quercetin (0.49–0.55 mg/g).

The consortium of capsaicinoids, carotenoids, and phenolic compounds (among others) is commonly used in the food, pharmaceutical, and cosmetics industry in the form of chili pepper oily extract, a high value ingredient called *Capsicum* oleoresin [25]. These natural liquid extract is renowned for inheriting all of the above mentioned properties and functional aspects of chili pepper bioactive content stored within the resin fraction, hence its common designation as “liquid spice” or as “chili pepper essence” [26]. In the food industry, this is used as food additive, specifically as flavor and color enhancers [27]. This additive can also be used to provide antimicrobial functional properties, preventing microbial growth and food spoilage, to ensure the final food product safety [28] and longer lifespan [29], as capsaicinoids do not degrade over time in normal storage conditions. The commercial and industrial appeal of this additive is mainly attributed to oleoresin’s pungency. Capsaicinoids’ spiciness factor is usually measured in Scoville Heat Units (SHU), a spiciness scale created by Wilbur Scoville in 1912 [30] with values ranging from 0 SHU (bell pepper, *Capsicum annuum* L.) up to 1,300,000 SHU (Carolina Reaper hot pepper, *Capsicum chinense* Jacq.) or even higher [31]. The spicier and more pungent the oleoresin, the higher the concentration of spicy compounds and thus the more functional and valuable the oleoresin.

Traditionally, the extraction of *Capsicum* oleoresin has relied on hexane, an organic solvent with environmental and health concerns. Several authors, such as Olguín-Rojas et al. [32], Yasin et al. [8], and Lu et al. [33] suggested that recovering *Capsicum* oleoresins from industrial by-products can be achieved using efficient, modern, and eco-friendly technologies to improve process and mitigate the associated environmental challenges. In recent decades, ultrasound technology has gained interest because it enables the rapid and resource efficient recovery of high value compounds [32,34,35]. This method has substantial evidence of scalability for industrial applications, although it has limitations regarding frequency and power, as underscored by Peshkovsky [36]. Nonetheless, when reviewing studies focusing on the extraction of *Capsicum* oleoresins from industrial chili pepper by-product using ultrasound technology, no optimal conditions have been identified that ensure maximum recovery of a high-quality oleoresin. Optimizing the extraction yield, and specifically of capsaicinoids and phenolic compounds, is relevant, as these compounds’ concentration directly impacts the antioxidant capacity registered in the final chili pepper oleoresin, with higher concentrations translating into higher antioxidant activity, as demonstrated by Johnson et al. [37]. Optimizing extraction conditions is strongly recommended, as there are several extraction related factors and quality parameters to consider. Ultrasound technology relies on critical parameters, such as extraction time, ultrasound power, solid to liquid ratio, temperature, particle size, type of solvent, and affinity, among others, with various impacts on the extract’s quality [38]. When optimized, these parameters can maximize process efficiency while adhering to sustainability principles, as demonstrated by Majid and Silva [39], who extracted antioxidants from manuka (*Leptospermum scoparium)* leaves using ultrasound technology. Numerous studies have explored the use of ultrasound technology for the extraction of valuable compounds from *Capsicum* fruits, but its application to *Capsicum* industrial by-products is scarce. As the interest in recovering value from agri-food wastes and by-products increases, the optimization of ultrasound assisted extraction (UAE) for the oleoresin extraction from *Capsicum* by-product represents an open field for research and innovation to enhance efficiency and extract quality while minimizing resources usage.

This study aimed to optimize the ultrasound assisted extraction of *Capsicum* oleoresin from industrial Red Habanero (*Capsicum chinense* Jacq.) chili pepper by-product to produce a valuable spicy, colorant, and bioactive food ingredient. 

The specific objectives were as follows:

1. Study the effects of UAE time and Acoustic Power Density (APD) on process efficiency parameters.
2. Study the effects of UAE time and APD on capsaicinoids, total phenolics, antioxidant activity, color, and peroxide value of the oleoresin produced.
3. Generate mathematical models for each response using experimental design and response surface methodology (RSM), and optimize UAE processing conditions.
4. Experimentally validate the responses for the predicted optimum ultrasound conditions.
5. Compare results obtained with optimized UAE conditions with reflux-assisted extraction (RAE), as described by AOAC for capsaicinoids extraction.

**Figure 1:**
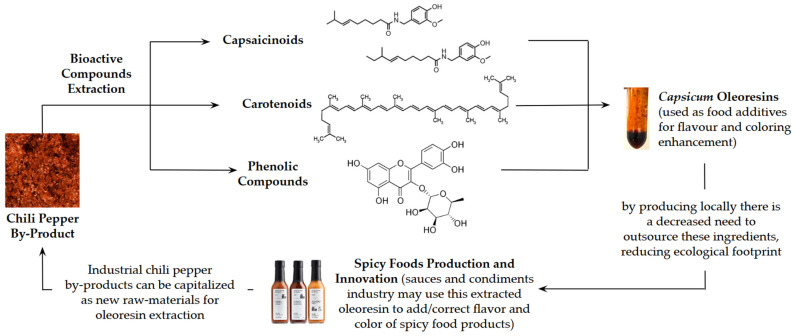
Circular pathway showcasing chili pepper by-product bioactive compounds extraction and transformation into a value-added *Capsicum* oleoresin.

Recovering these high-value extracts can uphold the principles of the circular economy by reintegrating them into companies’ value chains, reducing costs associated with outsourcing their acquisition, while prompting natural food products innovation (Figure 1), fostering localized production, and altogether promoting a more sustainable and responsible agro-industrial landscape [40,41].

## 2. Materials and Methods

### 2.1. Red Habanero Chili Pepper By-Product

**Figure 2:**
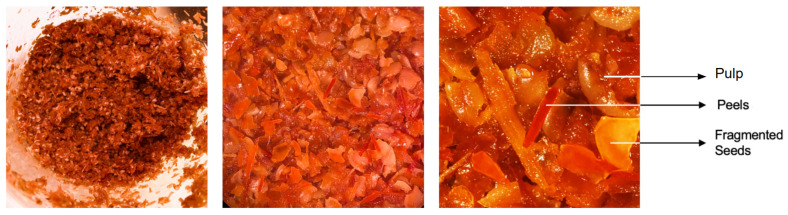
Photographs of industrial Red Habanero (*Capsicum chinense* Jacq.) chili pepper by-product and its main components (peels and fragmented seeds). Second and third photos are amplified versions of the first photograph shown on the left.

Industrial Red Habanero chili pepper (*Capsicum chinense* Jacq.) by-product was produced and provided by Casa Mendes Gonçalves (CMG, Portuguese condiments and seasonings producer) and kept frozen at −20 °C until extraction to preserve it and hinder any microbial or enzymatic degradative reactions. This by-product was composed by seeds, fibers, peels or skins, and residual pulp, with particles ranging from 4 to 10 mm, presenting a semi-solid consistency (Figure 2).

### 2.2. Experimental Design to Study the Effect of Acoustic Power Density and Ultrasound Extraction Time on Several Parameters

Response Surface Methodology (RSM) was employed using a central composite rotatable experimental design (CCDr) to optimize *Capsicum* oleoresin recovery from Red Habanero chili pepper by-product with ultrasound-assisted extraction (UAE). CCDr was chosen for being particularly effective for developing models for one or more response variables without the need to test all possible combinations of factors, thus conserving time and resources. The CCDr consisted of four factorial points, four center points, and four star points (Table 1).

Extraction time (3, 5, 10, 15, and 17 min) and Acoustic Power Density (APD of 0.30, 0.40, 0.65, 0.90 and 1.00 W/mL) were defined as independent variables. Ethanol (96% *v*/*v*) was used as the extraction solvent, as it is food grade and legal to use in the processing of raw materials and food ingredients according to Directive 2009/32/EC [42]. The best extraction conditions were selected based on the responses assessed: energy consumption (E), final temperature (T), and extraction yield (Y), alongside other *Capsicum* oleoresin relevant quality parameters such as capsaicinoids content (CAP), total phenolic content (TPC), antioxidant capacity (measured by FRAP and DPPH assays), peroxide value (PV), and color difference (∆E). The extraction time and APD were mathematically optimized to maximize the *Capsicum* oleoresin yield and bioactive content while minimizing temperature and energy consumption (attending to a more sustainable and economic industrial scenario). 

Predicted values for optimized ultrasound assisted extraction (UAE) conditions were experimentally determined and compared to those obtained from reflux-assisted extraction (RAE), both designed for *Capsicum* oleoresin extraction. 

**Figure 3:**
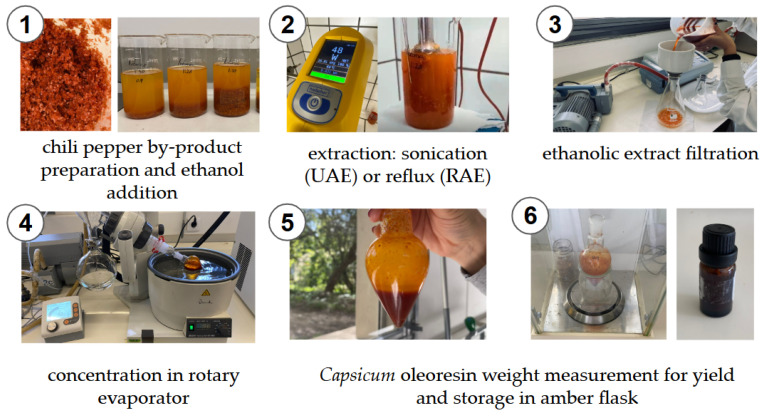
Illustrative scheme of the main steps for obtaining *Capsicum* oleoresin from industrial Red Habanero (*Capsicum chinense* Jacq.) by-product (numbers in the photographs indicate the sequence of the operations).

A scheme with overview of the main workflow steps to produce *Capsicum* oleoresin is shown in Figure 3.

### 2.3. Extraction Processes

The extraction processes used in this study were ultrasound assisted extraction (UAE) and reflux assisted extraction (RAE) for comparison between an innovative method and a traditional extraction method. The RAE extraction procedure followed the Association of Official Agricultural Chemists (AOAC) 995.03 method [43].

#### 2.3.1. Ultrasound Assisted Extraction (UAE)

Before extraction, 25 g of fresh by-product (corresponding to 7.5 g of dry matter) was weighed directly into a 200 mL glass beaker (15 cm height and 5 cm diameter) and 150 mL of 96% ethanol (v/v) was added to ensure a solid to liquid ratio of 1:20 on a dry basis. The ultrasound equipment (Hielscher UP200 Ht, Hielscher Ultrasound Technology GmbH, Teltow, Germany), consisting of a 200 W (26 kHz) head, was coupled with a 14 mm diameter titanium probe (Hielscher Sonotrode: S26d14, Hielscher Ultrasound Technology GmbH, Teltow, Germany). A digital thermometer and a power meter were used to monitor the temperature and energy consumption, respectively, in order to verify energy expenditure and compare its effects on oleoresin pungency and color across different experimental conditions. Sonication was carried out by setting the time (3, 5, 10, 15, and 17 min) and acoustic power density (0.3, 0.4, 0.65, 0.9, 1.0 W/mL) on the equipment’s interface after the titanium probe was submerged 5 cm deep in the mixture. The temperature probe was inserted almost entirely into the beaker, ensuring it did not touch the glass walls or the titanium probe. 

After sonication, the ethanol extracts were filtered, then concentrated using a rotary evaporator (Rotavapor® R II, Büchi®, BÜCHI Labortechnik AG, Flawil, Switzerland) equipped with a vacuum pump and a water bath set to 40 °C with condensers at −20 °C (Figure 3). A viscous oil referred to as *Capsicum* oleoresin was obtained and stored at 4 °C, shielded from direct light by using 10 mL amber glass flasks.

#### 2.3.2. Reflux Assisted Extraction (RAE)

RAE occurred for five hours at 80 °C, following the AOAC 995.03 [43] guidelines. Before extraction, 33.33 g of fresh by-product (corresponding to 10 g of dry matter) was weighed directly into a round-bottom flask, and 200 mL of 96% ethanol (v/v) was added to ensure a solid to liquid ratio of 1:20 on a dry basis. The flasks were placed in a heated mantle and coupled to cold water condensers to maintain solvent reflux. 

After extraction, the ethanolic extracts were filtered, concentrated until oleoresin was obtained, and stored at 4 °C, shielded from direct light in the same conditions mentioned in the previous section for UAE.

#### 2.3.3. Extraction Yield Determination

After concentration, the oleoresin was weighed to determine the extraction yield using Equation (1):

(1) $$Extraction\;Yield\;(\%)=\frac{Oleoresin\;weight\;\left(g\right)\times 100}{By-product\;weight\;(g,\;dry\;basis)}$$

### 2.4. Physicochemical Parameters Analysis

#### 2.4.1. Capsaicinoids

Capsaicinoids (CAP) quantification, specifically capsaicin and dihydrocapsaicin, was conducted using High Performance Liquid Chromatography (HPLC), following a modified version of the standardized AOAC method 995.03. The HPLC setup consisted of a diode array detector, an automatic thermostatic column compartment, and a column LC-C18 (150 × 4.6 mm i.d., 5 μm particle size). The samples were filtered using a 0.22 μm nylon filter and injected through a 20 μL loop in a mobile phase consisting of 60% acetonitrile and 40% Milli-Q water (with 1% acetic acid, v/v) at an isocratic flow rate of 1.0 mL/min. The column was kept at 40 °C, and each run took 18 min. Chromatograms generated from the analysis were processed using Chromeleon © Dionex software (Version 7.2.1.5833). As a standard, N-Vanillylnonanamide (NVA) was used in different concentrations (0.10, 0.25, 0.50, 0.75 and 1.00 mg/mL). The sum of the CAP values (capsaicin + dihydrocapsaicin, the main chemical players in spiciness) was expressed in milligrams of NVA equivalents. The Scoville Heat Units (SHU) scale was used to convert results into pungency levels (Equation (2)) to express the spiciness intensity in some cases.

(2) $$SHU=\frac{capsaicinoids\;\left(in\;ppm\right)\times 16}{sample\;weight\;(g,\;dry\;matter)}$$

#### 2.4.2. Total Phenolic Content

The total phenolic content (TPC) of the ethanolic extracts was determined using a modified Folin–Ciocalteu method used by Kupina et al. [44]. The procedure involved transferring 250 μL of each ethanolic extract (prior to concentration) into tubes, followed by the addition of 250 μL of 99.98% of ethanol (v/v), 1875 μL of distilled water, 250 μL of a 10% sodium carbonate solution (w/v), and 125 μL of a Folin–Ciocalteu reagent. After mixing, the samples were left at room temperature to react in the dark for one hour to allow the phenolic compounds to develop color through interaction with the Folin–Ciocalteu reagent. Following the incubation period, the absorbance of each sample was measured at a wavelength of 765 nm using a UV-visible spectrophotometer (Agilent Technologies, Cary 60 UV-Vis, Santa Clara, CA, USA) with distilled water serving as a blank. Each sample was analyzed in triplicate to ensure accuracy. A standard curve was prepared using different concentrations of gallic acid (0.02, 0.03, 0.05, 0.07, 0.08, 0.1, and 0.13 mg/mL). The final TPC values were expressed in milligrams of gallic acid equivalents (GAE) per gram of by-product on a dry basis or per gram of oleoresin.

#### 2.4.3. Antioxidant Activity

##### FRAP Assay

The Ferric Reducing Antioxidant Power (FRAP) assay was based on the Benzie and Strain [45] procedure. Briefly, a FRAP reagent was freshly prepared by mixing 30 mL of 0.3 M acetate buffer (pH 3.6), 3 mL of 20 mM ferric chloride hexahydrate (FeCl_3_·6H_2_O), and 3 mL of TPTZ solution (prepared in 40 mM HCl). This solution was stored at 4 °C, shielded from light, and used on the day of preparation. For the analysis, 90 μL of each extract was mixed with 270 μL of distilled water and 2700 μL of the FRAP reagent in 15 mL amber tubes. The samples were mixed and incubated at 37 °C in a water bath for 10 min. After incubation, the absorbance was measured at 593 nm using a UV-visible spectrophotometer (Agilent Technologies, Cary 60 UV-Vis, USA), with distilled water as the blank. A standard curve was prepared using different concentrations of FeSO_4_ (0.3, 0.4, 0.7, 1.0, 1.5, and 1.7 mM). The results were expressed in μmol of ferrous sulfate equivalents (FeSO_4_ eq) per milligram of by-product (dry basis) or per gram of oleoresin.

##### DPPH Radical Scavenging Activity Assay

The 2,2-diphenyl-1-picrylhydrazyl (DPPH) radical was prepared based on the Brand–Williams [46] procedure. Briefly, DPPH was prepared by dissolving 14.7 mg of DPPH radical in 25 mL of ethanol. For the analysis, 75 μL of each extract was mixed with 3000 μL of the DPPH radical in experimental tubes. After mixing, the samples were kept in the dark for 20 min. The absorbance was then measured at 515 nm using the same UV-visible spectrophotometer (Agilent Technologies, Cary 60 UV-Vis, USA), with ethanol serving as the blank. A standard curve was prepared using different concentrations of Trolox reagent (0.1, 0.3, 0.6, 0.8, 1.0, 1.2, and 1.4 mM). The results were expressed in μmol of Trolox Equivalent Antioxidant Activity (TEAC) per milligram of by-product on a dry basis or per gram of oleoresin.

#### 2.4.4. Diluted *Capsicum* Oleoresin Color Parameters

The oleoresin was diluted in sunflower oil at a 1:100 (w/w) ratio, mimicking industrial practices due its overhigh pungency and darkness, and the color parameters were measured by the CIELab color system (L* = lightness, a* = red-green axis, b* = yellow-blue axis) in a UV-Vis spectrophotometer (Agilent Technologies, Cary 60 UV-Vis, USA). The spectrophotometer was programmed to scan wavelengths between 380 and 780 nm at 5 nm intervals. A baseline correction was performed, using pure sunflower oil as the blank. Within the CIELab color system, a reading angle of 10°, a D65 illuminant (daylight), and transmittance (%T) measurements were configured. The diluted oleoresin samples were then placed in disposable cuvettes and analyzed three times to ensure accuracy. The color difference (ΔE) values (Equation (3)) indicate the difference in coloration between two samples. In this case, the higher ΔE values represent the higher presence/concentration of pigments in sunflower oil (used as the baseline). This difference is generally visually observed by the human eye when ΔE > 5, as indicated by Castellar et al. [47].

(3) $$\Delta E=\sqrt{{\left(L_{oil}-L_{oleoresin}\right)}^{2}+{\left(a_{oil}-a_{oleoresin}\right)}^{2}+{\left(b_{oil}-b_{oleoresin}\right)}^{2}}$$

#### 2.4.5. Peroxide Value

The peroxide value (PV) was determined using an adapted small-scale of AOCS Official Method Cd 8b-90 [48]. A 0.01 N sodium thiosulfate titrant was prepared and placed in a burette, alongside an isooctane/glacial acetic acid mixture (40:60, v/v). Then, a daily saturated potassium iodide (KI) solution and a starch indicator stored away from light were prepared. A 0.02 g sample of the obtained *Capsicum* oleoresin was added to 10 g of sunflower oil, then mixed with 50 mL of the isooctane/glacial acetic acid solution, homogenized, and 0.5 mL of KI was added. After shaking and resting in the dark for one minute, 50 mL of distilled water and 0.5 mL of the starch indicator were added. The solution was titrated with sodium thiosulfate until the darker color vanished, and the PV was calculated using Equation (4), expressed in meq O_2_/kg, as follows:

(4) $$PV\;(\frac{meqO_{2}}{kg})=\frac{Titrant\;volume\times 0.01\times 1000}{sample\;weight\;(g)}$$

### 2.5. Statistical Analysis

All experimental data were expressed as mean ± standard deviation (SD) from at least three independent replicates, unless specified otherwise. The statistical analysis for this study was divided into two main components: (1) Response Surface Methodology (RSM) using a Central Composite Experimental Design rotatable (CCDr), and (2) Analysis of variance (one-way ANOVA) for the comparison of the two extraction technologies performance. For RSM, the data were processed and modeled using Design Expert Version 12.0.0 (Software Stat-Ease Inc., Minneapolis, MN, USA) with the response variables modeled as functions of extraction time and APD. After completing the modeling, surface plots were obtained to better visualize the influence of a factor (variable) on each individual response, and the software’s point prediction tool was employed to estimate the optimal UAE conditions. For the comparison of the two extraction technologies, a statistical analysis of the experimental data was performed using STATISTICA software (StatSoft, Inc., StatSoft, version 13) using variance analysis (one-way ANOVA) and the Tukey test post hoc comparison at a significance level of 95% (*p* < 0.05).

## 3. Results and Discussion

### 3.1. Ultrasound-Assisted Extraction Conditions Responses and Impact on Oleoresin Quality

All of the results obtained gathered by using the Central Composite Design Rotatable (CCDr) approach are presented in Table 2. For Table 2, we considered as responses all those related to process efficiency (energy consumption, final temperature, and extraction yield), bioactive and antioxidant activities (capsaicinoids contents, total phenolic content, FRAP. and DPPH assays), and color assessment (*Capsicum* diluted oleoresin). The peroxide values are listed in Appendix A, but discussed in Section 3.1.4.

#### 3.1.1. Energy Consumption, Temperature, and Extraction Yield

Under UAE conditions of 15 min and APD of 0.90 W/mL (run 4), the highest energy consumption (33.92 ± 0.14 W·h) and the final temperature (82.67 ± 2.52 °C) were registered. In contrast, the minimum values were registered for UAE conditions of 3 min and an APD of 0.65 W/mL (run 10) for both energy consumption (4.92 ± 0.03 W·h) and temperature (33.67 ± 0.58 °C). As expected, the higher the energy consumed, the higher the final temperature reached by the extraction mixture. With respect to extraction yield, the highest value (26.54 ± 0.49%) was registered on run 11 (time: 10 min, APD: 1.00 W/mL) and the lowest (17.50 ± 0.36%) on run 2 (time: 5 min, APD: 0.40 W/mL). These results support that higher extraction times and higher energy inputs (resulting in higher temperatures) might not directly correspond to a higher increase in extraction yield [38]. In fact, lowering extraction times while maximizing yield might turn into industrial advantage, as excessive processing times could be economically unsustainable in the long term [39]. Furthermore, although temperature was not an independent parameter, values between 60 °C and 70 °C are ideal as capsaicinoids (related to spiciness) achieve fusion around 65 °C, facilitating their dissolution in the medium [7,33,34]. Thus, phenolic compounds are reported to be safely extracted around these temperatures without significant losses. Higher temperatures, although favoring extraction, might compromise sensible compound integrity, lowering the overall quality of the extract and final product as mentioned by Cárdenas-Castro et al. [49] and Elizalde-González [50].

#### 3.1.2. Capsaicinoids, Total Phenolic Content, and Antioxidant Activity of Oleoresin

For UAE conditions of 10 min and APD of 1.00 W/mL (run 11), both capsaicinoids (CAP) and total phenolic content (TPC) values were maximumized (CAP: 8.28 ± 0.54 mg NVA/g d.b.; TPC: 5.05 ± 0.43 mg GAE/g d.b.), while the highest antioxidant activity values (FRAP: 134.40 ± 3.49 μmol FeSO_4_ eq/g d.b.; DPPH: 32.64 ± 0.45 μmol TEAC/g d.b.) were registered on run 9 (time: 17 min, APD: 0.65 W/mL). Of all the responses, run 2 (time: 5 min, APD: 0.40 W/mL) showcased the lowest values (CAP: 3.60 ± 0.02 mg NVA/g d.b.; TPC: 1.90 ± 0.10 mg GAE/g d.b.; FRAP: 61.87 ± 0.59 μmol FeSO_4_ eq/g d.b.; DPPH: 6.33 ± 0.14 μmol TEAC/g d.b.). Overall, these results suggest that while longer extraction and higher APD can increase capsaicinoids and phenolic compound yields, they also risk reducing the overall antioxidant capacity after a certain point. This balance is key to preventing the degradation of sensitive bioactive compounds while maximizing their bioactive properties and molecular integrity. In fact, Atalay and İnanç [51] also detected that UAE may become hostile if used for longer periods, negatively impacting DPPH radical scavenging and the TPC of oleoresin from *Capsicum annuum* seeds by-product. This is likely due the fact that ultrasound equipment provides localized energy, rises in temperature and motion in the system, favoring oxidation and other degradative chemical reactions [52].

#### 3.1.3. Oleoresin Color Parameters

For the lightness parameter (L*), the highest value corresponding to a lighter color was observed in run 2 (91.26 ± 0.96) under UAE conditions of 5 min and an APD of 0.40 W/mL, while the lowest value, corresponding to a darker oleoresin, was observed in run 6 (78.94 ± 1.32) under 10 min and an APD of 0.65 W/mL. Regarding the chromatic parameter a* (redness), run 6 exhibited the highest value (18.63 ± 0.59), reflecting an intense red hue, whereas run 4 (15 min, 0.90 W/mL) resulted in the lowest value (10.38 ± 1.20). The b* parameter (yellowness) was most prominent in run 5 (76.05 ± 0.42), which corresponds to UAE conditions of 10 min and an APD of 0.65 W/mL. Conversely, the lowest b* value was registered in run 2 (56.63 ± 1.61). For the total color difference (ΔE), the highest value was observed in run 6 (77.25 ± 0.80) under UAE conditions of 10 min and an APD of 0.65 W/mL, indicating significant deviations in the original color. In contrast, the lowest ΔE value was recorded in run 2 (52.75 ± 1.51), showcasing minimal color changes under UAE conditions of 5 min and an APD of 0.40 W/mL. This RSM data suggests that the combination of higher extraction times with higher APDs lead to adverse effects in oleoresin coloration, suggesting some carotenoid content loss. Such elevated temperatures (resulting from higher energy inputs) could lead to pigment loss or degradation, thus affecting the overall color profile of the extracted oleoresin. Vulić et al. [21] and Giuffrida et al. [18] both stated that *Capsicum* pigments are extremely unstable in their free form, being more susceptible to oxidation. Previous studies [32,51] showed that if correctly used, UAE provides higher pigment extraction rates when compared to other technologies such as supercritical fluid extraction, maceration, and even conventional solvent extraction, providing the best results in less time and requiring less energy. This highlights the importance of optimizing extraction conditions to prevent any type of degradation of valuable pigments during the extraction process.

#### 3.1.4. Peroxide Value

Given the oily nature of *Capsicum* oleoresin, peroxide value (PV) is a good indicator of oil degradation. In theory, ultrasound cavitation can promote oxidative reactions, with generation of oxidative radicals such as peroxides and hydroperoxides, as mentioned by Marhamati et al. [53]. Analyzing the RSM data, PV (available in Appendix A) did not vary substantially across different UAE conditions (0.25 ± 0.00 to 0.55 ± 0.26 meq O_2_/kg). Moreover, the PV results were extremely low and similar among different extraction treatment conditions, suggesting negligible oil alteration by sonication. In this way, PV might be independent from extraction time and APD in these studied conditions. These study results differ from those found in the study of Atalay and İnanç [51], in which sonicated oleoresin obtained from pepper seeds presented higher PVs when compared to a non-sonicated control. This difference might be attributed to the lower extraction times applied in this study (3–17 min) when compared to those applied by the cited authors (20–40 min), as higher extraction times promote higher degradation reactions to occur in the long run.

### 3.2. Mathematical Models and Surface Plots for Each Parameter

Table 3 shows the correlation (R^2^) and the equation for the second order model obtained from the statistical analysis of each individual response value derived from CCDr. Each response was adjusted to a second order mathematical model (except energy consumption, where a first order model was more appropriate), presenting R^2^ values equal to or above 0.90 for all responses. PV response is not shown due to a lack of correlation (R^2^ < 0.20), suggesting no effect of extraction time and APD on this quality parameter. With respect to the lack of fit test, the energy consumption and total phenolic content parameters were well fitted (see Supplementary Materials for ANOVA results). However, all of the other responses revealed a statistically significant lack of fit (p < 0.05), indicating potential issues with the mathematical models. Real experiments were carried out for optimum extraction conditions (time and APD) to further validate the prediction capacity of the mathematical models, as shown in the following section.

**Figure 4:**
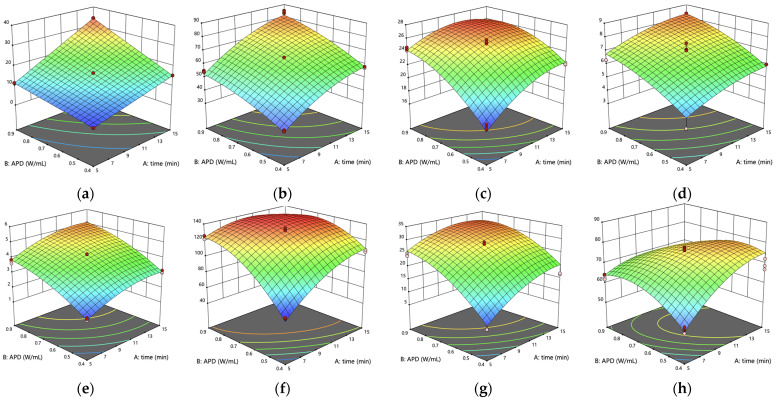
3D response surface plots showing the effects of time (min) and APD (W/mL) on individual responses: (**a**) E = energy consumption (W·h); (**b**) T = final temperature (°C); (**c**) Y = extraction yield (%, d.b.); (**d**) CAP = capsaicin + dihydrocapsaicin content (mg NVA/g, d.b.); (**e**) TPC = total phenolic content (mg GAE/g, d.b.); (**f**) FRAP = ferric reducing antioxidant power (μmol FeSO_4_ eq/g, d.b.); (**g**) DPPH (μmol TEAC/g, d.b.); (**h**) Oleoresin color variation (ΔE).

Figure 4 displays the response surface plots illustrating responses dependence on extraction time and APD factors.

These 3D plots effectively capture the interactions between variables, which suggest that while moderate conditions optimize extraction, excessive processing may lead to a plateau in yield and a decline in the antioxidant properties of the extract. Starting with energy consumption, the model showed a perfect fit, revealing that both extraction time and APD significantly affect energy use. The final process temperature, closely related to energy consumption, followed a more quadratic relation, where longer times and higher APDs increased both energy and temperature up to a threshold of around 80 °C. While higher temperatures can improve yield by enhancing cellular breakdown, they also risk degrading sensitive compounds like capsaicinoids and phenolics [50]. The extraction yield model highlighted a threshold beyond which further increases in time and APD lead to diminishing returns, likely due to solvent saturation and the depletion of extractable materials, which is observable by the decreasing antioxidant activity registered by FRAP and DPPH after a certain point in time (>10 min) and APD (>0.90 W/mL). The color intensity in pepper by-product and diluted oleoresin also indicated the effects of extraction conditions on pigment and bioactive compound integrity, with color serving as a potential indicator of over-processing as reported by Rafajlovska et al. [54]. These quadratic models, overall, support the statement that beyond a certain time and APD in given conditions, ultrasound-assisted extraction positive effects reverse. Some of the differences are possible to see within the experimental conditions range. Others peak somewhere beyond our experimental bounds and may be observable using longer extraction times and a more powerful ultrasound equipment.

### 3.3. Experimental Validation of Optimum Ultrasound-Assisted Extraction (UAE) Conditions and Comparison with Reflux-Assisted Extraction (RAE)

The results of the model’s predicted responses’ confidence intervals (95%), the corresponding results obtained experimentally, and the results of using reflux-assisted extraction are shown in Table 4. Using the response models obtained in the previous section, it was possible to mathematically calculate the optimum conditions for maximizing oleoresin quality. The optimum ultrasound-assisted extraction (UAE) conditions (mathematically determined by Design Expert software using RSM data) for recovering *Capsicum* oleoresin from chili pepper by-product with the highest yield and bioactive content were 8 min of extraction time and 0.87 W/mL of APD, maximizing quality while accomplishing high energy efficiency, ultimately lowering energy-related production costs. For these optimized conditions, all of the responses were predicted and then tested in real experiments.

For each response, the experimental values fit within their respective confidence intervals, which confirms that despite the statistically significant lack of fit for some of the parameters’ equations, the prediction capacity is reliable within the design space investigated. When compared to the literature, the optimized extraction yield (Y) in this study (26.03%) is supported by both the predicted and experimental values and is higher than those typically reported for oleoresins obtained from chili pepper by-product using sonication [22,32,35], only being surpassed by Olguín-Rojas et al. [32] (31.49%). This success can be attributed to UAE’s ability to retain not only target compounds like capsaicinoids and pigments but also other matrix components such as sugars and salts [3,9], increasing the final extract mass. While these additional components may not directly enhance bioactivity, they contribute to the overall yield, making UAE economically appealing for food industries aiming to maximize extraction yields. Furthermore, the extraction required 8 min for a maximized completion, which is favorable from an industrial standpoint as unnecessary prolonged extraction times are economically impractical from the industry’s perspective [39].

The optimized capsaicinoids (CAP) content achieved in this study (7.47 mg/g) was lower than the 10.64 mg/g reported by Olguín-Rojas et al. [32] for the same Red Habanero (*Capsicum chinense* Jacq.) by-product variety. This difference may be attributed to biological variations in the pepper fruit, the use of different solvents, by-product composition, or the absence of a drying process in the present study. However, the spiciness level, rather than the absolute CAP content, is generally the primary factor of interest in *Capsicum* oleoresin. The oleoresin obtained under optimal UAE conditions demonstrated a spiciness of approximately 466,000 Scoville Heat Units (SHU), equivalent to consuming 2 fresh Red Habanero fruits or 50 fresh Jalapeños, using the research of Orellana-Escobedo et al. [55] as reference. In industry terms, commercial oleoresins available in the market are benchmarked with 500,000 SHU, 1,000,000 SHU, and 1,500,000 SHU, which demonstrates that the oleoresin obtained from Red Habanero by-product in this present study is closely related, capable of serving the same industrial role. This level of spiciness makes the extract suitable for imparting mild to extreme heat in a wide range of food products [25], which means it is still useful in a food industry context. Remarkably, the CAP content in the oleoresin from industrial Red Habanero by-product (29.12 mg/g of oleoresin) exceeded the value reported for fresh Red Habanero fruits (24.86 mg/g of oleoresin) by Valencia-Cordova et al. [29].

On the other hand, the optimized total phenolic content (TPC) values (obtained from chili pepper by-product) were slightly lower than those obtained by the previously cited authors (present study: 17.26 mg GAE/g of oleoresin vs. literature: 29.13 mg GAE/g of oleoresin). Their oily extracts were produced by heating macerated Red Habanero peppers for 15 min at 65 °C with a 1:9 dry sample-to-solvent ratio. In contrast, the present study utilized a higher dry weight-to-solvent ratio (1:20) and a shorter extraction time (8 min), plus leveraging the cavitation effect inherent to UAE, which enhances chemical compound extraction. These factors not only enhanced the spiciness (surpassing even extracts derived from fresh peppers), they also likely preserved antioxidant activity levels (126.66 µmol TEAC/g of oleoresin) higher than the antioxidant activity reported by Valencia-Cordova et al. [29] for fresh Red Habanero oleoresin (11.30 µmol TEAC/g of oleoresin), demonstrating the efficiency of UAE in maximizing compounds extraction.

From an operational standpoint, optimized UAE conditions offer significant advantages over non-fully optimized extraction methods. The optimized extraction time (8 min) was shorter than those reported in previous studies, reducing energy consumption (17.44 ± 0.06 W·h) and overall processing costs. The optimized UAE time for Red Habanero by-product in this study was slightly shorter than the 10 min proposed as a new rapid method by Barbero et al. [34] for fresh cayenne pepper varieties (*Capsicum annuum* L.). This difference likely arises from the more powerful ultrasound equipment employed in this present study, as Barbero et al. used ultrasound water baths with lower energy output and higher frequency levels, thereby requiring longer extraction times to achieve proportionally higher yield levels. In an enlarged perspective, the optimized extraction settings in this present work (8 min and 0.87 W/mL APD) differ for those obtained for extraction of antioxidant from manuka leaves (14 min and 0.52 W/mL APD using 56% ethanol as solvent) and *Aristotelia serrata* leaves (20 min and 0.55 W/mL APD using 73% ethanol as solvent), both tested by Majid and Silva [39], where longer extraction times and lower APDs for optimum extraction of antioxidants were determined. Nonetheless, by performing extraction in less time using larger sample quantities (as demonstrated in our study), might turn into an advantage, as these factors better approach industrial priorities of extraction cost savings at a large scale. Furthermore, the larger presence of bioactive compounds such as capsaicin, dihydrocapsaicin, phenolic acids, and flavonoids enhance the overall antioxidant capacity of these oily additives, rendering more preservative and stabilizing properties to food products when incorporated, which is one of the main roles of *Capsicum* oleoresins in the food industry, as mentioned by Gallego [56].

These UAE optimized experimental values were then statistically compared to those obtained from the reflux-assisted extraction (RAE) (Table 4). UAE’s significant energy savings make it a more environmentally friendly option compared to traditional methods, aligned with the main ultrasound benefits described in the literature [38,52]. Overall, UAE exhibits higher performance when compared to RAE (α = 0.05), except for energy consumption and final temperature. There was 95% less energy consumption in UAE (17.44 ± 0.06 W·h compared to RAE (387.87 ± 0.29 W·h), without impairing the extraction of capsaicinoids, phenolics, and pigments from Red Habanero by-product. The nearly 95% reduction in energy demonstrates the efficiency of UAE, making it a much more energy-conscious option, especially relevant in large-scale operations where cost and energy use are critical [38,39]. Thus, the cavitation effect enhanced the extraction yield from nearly 21% (RAE) up to approximately 26% (UAE), a statistically significant improvement of 5% using the same solvent amount but requiring less energy and time. In fact, these results underline expectations, as ultrasound technology is commonly referred to as being energetically efficient over conventional methods and even novel extractive methods (e.g., microwaves, pulsed electric fields, supercritical fluids, etc.) for performing in less time and requiring less resources, as verified by Lu et al. [33] and Majid and Silva [39].

Regarding CAP, in just 8 min UAE promotes the recovery of extracts with the same spiciness (approximately 466,000 SHU) as those obtained by RAE during 5 h of extraction. In advantage, the TPC values in sonicated ethanolic extracts (4.29 ± 0.26 mg GAE/g by-product d.b.) are statistically higher than those obtained in RAE (2.41 ± 0.04 mg GAE/g by-product d.b.), likely due to cavitation extraction promoting cell wall disruption and higher solvent penetration rate [52]. In addition, the antioxidant activity of ethanolic extracts obtained by UAE were statistically higher (FRAP: 138.77 ± 3.84 μmol FeSO_4_ eq/g of by-product d.b.; DPPH: 32.91 ± 2.88 μmol TEAC/g of by-product d.b.) when compared to RAE (FRAP: 115.06 ± 4.51 μmol FeSO_4_ eq/g of by-product d.b.; DPPH: 12.33 ± 0.62 μmol TEAC/g of by-product d.b.), further supporting that UAE optimized conditions allow the extraction of more bioactive enriched extracts as mentioned in the explored literature [32,51]. Focusing on the color parameters of *Capsicum* oleoresin (diluted in sunflower oil), a commercial and industrial quality assessment practice, UAE promoted the recovery of the reddest oleoresin (UAE: a* = 15.58 ± 1.68 vs. RAE: a* = 8.45 ± 3.02), with the lowest lightness (UAE: L* = 81.06 ± 2.26 vs. RAE: L* = 90.22 ± 3.75), with darker coloration suggesting higher contents of beta-carotene, capsanthin, and capsorubin, all present in the majority of fruits from *Capsicum* species [15].

Altogether, oleoresin provided by UAE from chili pepper by-product is still suitable for healthy food innovation, aligned with consumer trends, and enhancing food’s overall quality and functionality. As demonstrated, these extracts can be sourced from what would be considered a “wasted by-product”, and its natural profile is ideal to provide additive functional capabilities to new natural food product formulations under the clean-label concept [57].

## 4. Conclusions

In conclusion, this study demonstrates that Ultrasound Assisted Extraction (UAE) is a highly efficient and sustainable method for the valorization of Red Habanero by-product into high-value oleoresin. The optimized UAE conditions, requiring only 8 min of extraction time and an acoustic power density (APD) of 0.87 W/mL, achieved the maximum extraction yield while preserving the bioactivity, reddish color, and spiciness of the oleoresin. The mathematical models generated in this study (e.g., response surface methodology) accurately predicted all of the real values for each parameter, despite the statistically significant lack of fit test for some of the parameters investigated. The cavitation effect inherent to UAE played a pivotal role in enhancing compound recovery and minimizing degradation. Additionally, it significantly reduced energy consumption by up to 95% compared to traditional reflux assisted extraction (RAE). The oleoresin obtained under these optimized conditions demonstrated remarkable properties, including a spiciness of approximately 466,000 Scoville Heat Units (SHU), superior antioxidant activity, and high red color intensity. These attributes make it suitable for various industrial applications, ranging from food seasoning to nutraceutical products. Furthermore, UAE’s superior efficiency and reduced environmental impact—through lower energy consumption and efficient solvent use—highlight its potential for large-scale use in circular economy strategies. Future research should explore the application of this method to other chili by-product varieties beyond the Red Habanero and investigate the integration of UAE with other green extraction methods, such as supercritical CO_2_ extraction, to verify the reproducibility and efficiency of this process across different plant matrixes.

## Figures and Tables

**Table 1:** Central Composite Design Rotatable (CCDr) coded and decoded factors considering time (min) as factor A and acoustic power density (APD, W/mL) as factor B.

	Factorial Points				Central Points				Star Points			
Run	1	2	3	4	5	6	7	8	9	10	11	12
A	−1	−1	1	1	0	0	0	0	α	−α	0	0
B	1	−1	−1	1	0	0	0	0	0	0	α	−α
time	5	5	15	15	10	10	10	10	17	3	10	10
APD	0.90	0.40	0.40	0.90	0.65	0.65	0.65	0.65	0.65	0.65	1.00	0.30

Coded values for factors A and B for CCDr experimental design are listed in the Table as −1, 0, 1, −α and α. The last two rows of the Table show the real values of variables time and APD corresponding to those codes.

**Table 2:** Ultrasound process efficiency parameters, capsaicinoid content, total phenolic content, antioxidant activity, and color parameters of oleoresin obtained from Red Habanero by-product for different extraction conditions according to a Central Composite Design Rotatable (CCDr).

Run	UAE Conditions		Responses			
	Time (min)	APD (W/mL)	Efficiency Parameters			
			E (W·h)	T (°C)	Y (%)	
01	5	0.90	11.37 ± 0.07	54.67 ± 0.58	24.40 ± 0.24	
02	5	0.40	5.06 ± 0.05	34.33 ± 0.58	17.50 ± 0.36	
03	15	0.40	15.25 ± 0.00	57.67 ± 0.58	22.22 ± 0.16	
04	15	0.90	33.92 ± 0.14	82.67 ± 2.52	25.16 ± 0.36	
05	10	0.65	16.39 ± 0.10	64.33 ± 0.58	25.13 ± 0.16	
06	10	0.65	16.39 ± 0.10	63.67 ± 0.58	25.36 ± 0.47	
07	10	0.65	16.39 ± 0.10	64.67 ± 0.58	25.17 ± 0.16	
08	10	0.65	16.39± 0.10	64.00 ± 0.00	25.21 ± 0.24	
09	17	0.65	27.86 ± 0.16	73.0 ± 1.00	25.67 ± 0.41	
10	3	0.65	4.92 ± 0.03	33.67 ± 0.58	19.29 ± 0.19	
11	10	1.00	25.06 ± 0.10	71.33 ± 0.58	26.54 ± 0.49	
12	10	0.30	7.56 ± 0.10	43.67 ± 1.15	18.23 ± 0.37	
Run	UAE Conditions		Responses			
	Time (min)	APD (W/mL)	Chemical parameters			
			CAP	TPC	FRAP	DPPH
01	5	0.90	6.31 ± 0.01	3.74 ± 0.13	122.91 ± 2.33	24.38 ± 0.61
02	5	0.40	3.60 ± 0.02	1.90 ± 0.10	61.87 ± 0.59	6.33 ± 0.14
03	15	0.40	5.98 ± 0.00	3.06 ± 0.08	106. 99 ± 1.49	16.57 ± 0.19
04	15	0.90	8.14 ± 0.22	5.00 ± 0.02	118.03 ± 0.78	27.52 ± 0.14
05	10	0.65	7.29 ± 0.36	4.20 ± 0.03	132.73 ± 1.78	28.38 ± 0.76
06	10	0.65	7.02 ± 0.12	4.20 ± 0.03	131.43 ± 0.94	27.17 ± 0.29
07	10	0.65	6.94 ± 0.10	4.21 ± 0.02	131.99 ± 0.38	27.92 ± 0.29
08	10	0.65	6.71 ± 0.30	4.21 ± 0.02	131.01 ± 1.29	27.87 ± 0.81
09	17	0.65	7.29 ± 0.02	4.31 ± 0.03	134.40 ± 3.49	32.64 ± 0.45
10	3	0.65	6.04 ± 0.06	2.56 ± 0.11	87.63 ± 1.12	16.47 ± 0.24
11	10	1.00	8.28 ± 0.54	5.05 ± 0.43	133.81 ± 1.74	30.78 ± 0.49
12	10	0.30	4.90 ± 0.25	2.07 ± 0.06	70.52 ± 0.59	12.94 ± 0.45
Run	UAE Conditions		Responses			
	Time (min)	APD (W/mL)	Color parameters			
			L*	a*	b*	ΔE
01	5	0.90	86.51 ± 1.23	12.90 ± 2.59	65.68 ± 1.00	62.92 ± 1.22
02	5	0.40	91.26 ± 0.96	10.55 ± 0.63	56.63 ± 1.61	52.75 ± 1.51
03	15	0.40	84.62 ± 0.88	14.47 ± 1.53	71.52 ± 2.29	69.20 ± 2.70
04	15	0.90	90.53 ± 1.27	10.38 ± 1.20	59.77 ± 4.64	55.85 ± 4.50
05	10	0.65	80.90 ± 0.96	16.76 ± 1.39	76.05 ± 0. 42	74.96 ± 0.50
06	10	0.65	78.94 ± 1.32	18.63 ± 0.59	77.42 ± 0.78	77.25 ± 0.80
07	10	0.65	80.64 ± 1.11	17.89 ± 1.73	76.50 ± 0.96	75.74 ± 1.61
08	10	0.65	82.45 ± 2.50	16.97 ± 0.92	76.46 ± 1.95	75.01 ± 1.97
09	17	0.65	88.80 ± 0.76	12.66 ± 2.32	62.31 ± 1.09	59.16 ± 1.32
10	3	0.65	89.65 ± 0.79	12.11 ± 0.62	59.29 ± 1.44	55.97 ± 1.19
11	10	1.00	88.04 ± 1.14	13.55 ± 1.15	76.89 ± 0.40	73.41 ± 0.72
12	10	0.30	88.61 ± 1.51	12.67 ± 0.57	62.84 ± 0.32	59.68 ± 0.72

Note: UAE = ultrasound-assisted extraction; APD = acoustic power density; E = energy consumption (W·h); T = final temperature (°C); Y = extraction yield (%, dry basis); CAP = capsaicin + dihydrocapsaicin content (mg NVA/g, dry basis); TPC = total phenolic content (mg GAE/g, dry basis); FRAP = ferric reducing antioxidant power (μmol FeSO_4_ eq/g, dry basis); DPPH (μmol TEAC/g, dry basis); L* = lightness axis; a* = (+) red/(−) green axis; b* = (+) yellow/(−) blue axis; ΔE represents the oleoresin (diluted in sunflower oil) color variation value which uses pure sunflower oil as base line: L*: 100 ± 0.00, a*: −1.94 ± 0.00, b*: 6.36 ± 0.01. All responses are presented as the mean ± standard deviation of three real replicates (n = 3).

**Table 3:** Equations for each response obtained by regression from the CCDr experimental design.

Responses	Units	R^2^	Prediction Equation
Energy Consumption	(W·h)	1.00	–0.19 + 0.03(A) + 0.29(B) + 2.47 (AB)
Final Temperature	°C	0.99	–23.49 + 6.07(A) + 92.54(B) + 0.93(AB) − 0.20(A^2^) − 45.70(B^2^)
Extraction Yield	% (d.b.)	0.97	–6.12 + 2.01(A) + 49.11(B) − 0.79(AB) − 0.06(A^2^) − 23.34(B^2^)
Capsaicinoids Content	mg NVA/g (d.b.)	0.87	–1.41 + 0.41(A) + 12.38(B) − 0.01(A^2^) − 5.79(B^2^)
Total Phenolic Content	mg GAE/g (d.b.)	0.98	–3.59 + 0.45(A) + 11.17(B) − 0.02(A^2^) − 5.50(B^2^)
FRAP	μmol FeSO_4_ eq/g, (d.b.)	0.97	–167.35 + 18.40(A) + 515.15(B) − 10.00(AB) − 0.46(A^2^) − 256.91(B^2^)
DPPH	μmol TEAC/g, (d.b.)	0.90	–46.15 + 4.25(A) + 119.10(B) − 1.42(AB) − 0.12(A^2^) − 58.09(B^2^)
Color Variation (ΔE)	—	0.92	–28.78 + 1.98(A) + 70.36(B) − 0.05(A^2^) − 37.16(B^2^)

Note: A = time (min); B = acoustic power density, APD (W/mL); ΔE represents the oleoresin (diluted in sunflower oil) color difference in relation to pure sunflower oil as base line: L*: 100 ± 0.00, a*: −1.94 ± 0.00, b*: 6.36 ± 0.01. Factors were excluded when *p*-value > 0.05 (α = 0.05).

**Table 4:** Comparison of predicted and experimentally obtained results for optimum ultrasound-assisted extraction (UAE) conditions, and conventional reflux assisted extraction (RAE).

	Ultrasound Optimized Extraction Conditions (Time: 8 min|APD: 0.87 W/mL)		Reflux Extraction (Time: 300 min|85 °C)
Responses	Predicted Confidence Interval (95%)	Experimental Values *	Experimental Values *
E (W·h)	[17.39–17.63]	17.44 ± 0.06 a	387.87 ± 0.29 b
T (°C)	[62.64–66.74]	66.00 ± 1.00 a	82.63 ± 3.72 b
Y (%)	[25.14–26.60]	26.03 ± 0.65 a	20.88 ± 0.66 b
CAP (mg NVA/g d.b.)	[6.66–8.19]	7.47 ± 0.32 a	7.61 ± 0.64 a
TPC (mg GAE/g d.b.)	[4.32–4.69]	4.29 ± 0.26 a	2.41 ± 0.04 b
FRAP (μmol FeSO_4_ eq/g d.b.)	[128.86–140.05]	138.77 ± 3.84 a	115.06 ± 4.51 b
DPPH (μmol TEAC/g d.b.)	[26.42–33.29]	32.91 ± 2.88 a	12.33 ± 0.62 b
Color Variation (ΔE)	[62.67–76.69]	75.38 ± 3.95 a	65.86 ± 0.88 b

Note: UAE = ultrasound-assisted extraction; APD = acoustic power density; E = energy consumption; T = final temperature; Y = extraction yield; CAP = capsaicin + dihydrocapsaicin content; TPC = total phenolic content; ΔE represents the oleoresin (diluted in sunflower oil) color difference using pure sunflower oil as reference. * All responses are presented as the mean ± standard deviation of three replicates (n = 3). Different letters in the same row indicate that the means differ significantly by Tukey’s test (*p* < 0.05).

## Data Availability

The original contributions presented in this study are included in the article/Supplementary Materials. Further inquiries can be directed to the corresponding authors.

## Supplementary Materials

The following supporting information can be downloaded at: https://www.mdpi.com/article/10.3390/foods14081407/s1, Table S1. Energy Consumption ANOVA results. Table S2. Energy Consumption model fit statistics. Table S3. Final Temperature ANOVA results. Table S4. Final Temperature model fit statistics. Table S5. Extraction Yield ANOVA results. Table S6. Extraction yield model fit statistics. Table S7. Capsaicinoid Content ANOVA results. Table S8. Capsaicinoid Content model fit statistics. Table S9. Total Phenolic Content ANOVA results. Table S10. Total Phenolic Content model fit statistics. Table S11. Antioxidant Activity: FRAP ANOVA results. Table S12. Antioxidant Activity: FRAP model fit statistics. Table S13. Antioxidant Activity: DPPH ANOVA results. Table S14. Antioxidant Activity: DPPH model fit statistics. Table S15. Diluted Oleoresin Color Variation ANOVA results. Table S16. Diluted Oleoresin Color Variation model fit statistics.

## Appendix A

Run:	1	2	3	4	5	6
Time (min)	5	5	15	15	10	10
APD (w/mL)	0.90	0.40	0.40	0.90	0.65	0.65
PV (meqO_2_/kg)	0.55 ± 0.26	0.41 ± 0.04	0.57 ± 0.34	0.41 ± 0.09	0.30 ± 0.07	0.43 ± 0.30
Run:	7	8	9	10	11	12
Time (min)	10	10	17	3	10	10
APD (w/mL)	0.65	0.65	0.65	0.65	1.00	0.30
PV (meqO_2_/kg)	0.31 ± 0.03	0.31 ± 0.21	0.25 ± 0.00	0.43 ± 0.17	0.50 ± 0.29	0.37 ± 0.03
Note: APD = acoustic power density; PV = Peroxide value. All responses are presented as the mean ± standard deviation of three real replicates (n = 3).						
