# Supplementary material for: Spicy Food Ingredient from Red Habanero By-Product Obtained by Ultrasound-Assisted Extraction
Source: Foods. 2025 Apr 18;14(8):1407. doi: 10.3390/foods14081407 (PMC12026765; doi:10.3390/foods14081407)
Supplement: Supplementary file 1 [file foods-14-01407-s001.zip › foods-3519497-supplementary.pdf]

Supplementary Statistical Tables

Manuscript: Spicy Food Ingredient From Red Habanero By-Product Obtained By Ultrasound-Assisted Extraction

Response Energy Consumption (E)

Table S1. Energy Consumption ANOVA results

Source	Sum of Squares	df	Mean Square	F-value	p-value	
**Model**	2635.81	3	878.60	1.071E+05	< 0.0001	significant
A-time	1593.92	1	1593.92	1.943E+05	< 0.0001	
B-APD	927.50	1	927.50	1.130E+05	< 0.0001	
AB	114.39	1	114.39	13942.52	< 0.0001	
**Residual**	0.2625	32	0.0082			
Lack of Fit	0.0375	5	0.0075	0.8992	0.4958	not significant
Pure Error	0.2251	27	0.0083			
**Cor Total**	2636.07	35				

Table S2. Energy Consumption model fit statistics

**Std. Dev.**	0.0906		**R^2**	0.9999
**Mean**	16.38		**Adjusted R^2**	0.9999
**C.V. %**	0.5531		**Predicted R^2**	0.9999

Model for *Energy Consumption (kW.h)* in terms of actual factors:

$$E = -0.19 + 0.03(\text{time}) + 0.29(\text{APD}) + 2.47\,(\text{time.APD})$$

Response Final Temperature (T)

Table S3. Final Temperature ANOVA results

Source	Sum of Squares	df	Mean Square	F-value	p-value	
**Model**	7515.55	5	1503.11	631.36	< 0.0001	significant
A-time	4288.24	1	4288.24	1801.22	< 0.0001	
B-APD	2676.97	1	2676.97	1124.43	< 0.0001	
AB	16.33	1	16.33	6.86	0.0137	
A^2	463.40	1	463.40	194.64	< 0.0001	
B^2	152.31	1	152.31	63.98	< 0.0001	
**Residual**	71.42	30	2.38			
Lack of Fit	47.09	3	15.70	17.42	< 0.0001	significant
Pure Error	24.33	27	0.9012			
**Cor Total**	7586.97	35				

Table S4. Final Temperature model fit statistics

**Std. Dev.**	1.54	**R^2**	0.9906
**Mean**	58.97	**Adjusted R^2**	0.9890
**C.V. %**	2.62	**Predicted R^2**	0.9852
		**Adeq Precision**	76.3560

Model for *Final Temperature (°C)* prediction in terms of actual factors:

$$T = -23.49 + 6.07(\text{time}) + 92.54(\text{APD}) + 0.93(\text{time.APD}) - 0.20(\text{time}^2) - 45.70(\text{APD}^2)$$

Response Extraction Yield (Y)

Table S5. Extraction Yield ANOVA results

Source	Sum of Squares	df	Mean Square	F-value	p-value	
**Model**	329.74	5	65.95	218.68	< 0.0001	significant
A-time	78.59	1	78.59	260.59	< 0.0001	
B-APD	174.80	1	174.80	579.62	< 0.0001	
AB	11.74	1	11.74	38.93	< 0.0001	
A^2	37.14	1	37.14	123.16	< 0.0001	
B^2	39.74	1	39.74	131.77	< 0.0001	
**Residual**	9.05	30	0.3016			
Lack of Fit	6.45	3	2.15	22.36	< 0.0001	significant
Pure Error	2.60	27	0.0962			
**Cor Total**	338.79	35				

Table S6. Extraction yield model fit statistics

**Std. Dev.**	0.5492		**R^2**	0.9733
**Mean**	23.32		**Adjusted R^2**	0.9688
**C.V. %**	2.35		**Predicted R^2**	0.9583
			**Adeq Precision**	41.6043

Model for *Extraction Yield (%)* prediction in terms of actual factors:

$$Y = -6.12 + 2.01(\text{time}) + 49.11(\text{APD}) - 0.79(\text{time.APD}) - 0.06(\text{time}^2) - 23.34(\text{APD}^2)$$

Response Capsaicinoid Content (CAP)

Table S7. Capsaicinoid Content ANOVA results

Source	Sum of Squares	df	Mean Square	F-value	p-value	
**Model**	34.72	4	8.68	40.40	< 0.0001	significant
A-time	9.02	1	9.02	41.96	< 0.0001	
B-APD	23.22	1	23.22	108.09	< 0.0001	
A^2	1.32	1	1.32	6.12	0.0229	
B^2	1.63	1	1.63	7.60	0.0126	
**Residual**	4.08	19	0.2149			
Lack of Fit	3.09	4	0.7730	11.71	0.0002	significant
Pure Error	0.9902	15	0.0660			
**Cor Total**	38.80	23				

Table S8. Capsaicinoid Content model fit statistics

**Std. Dev.**	0.4635		**R^2**	0.8948
**Mean**	6.54		**Adjusted R^2**	0.8726
**C.V. %**	7.08		**Predicted R^2**	0.8155
			**Adeq Precision**	18.5780

Model for *Capsaicinoid Content (mg NVA/g)* prediction in terms of actual factors:

$$CAPS = -1.41 + 0.41(time) + 12.38(APD) - 0.01(time^2) - 5.79(APD^2)$$

Response Total Phenolic Content (TPC)

Table S9. Total Phenolic Content ANOVA results

Source	Sum of Squares	df	Mean Square	F-value	p-value	
**Model**	37.39	4	9.35	490.79	< 0.0001	significant
A-time	8.98	1	8.98	471.67	< 0.0001	
B-APD	23.94	1	23.94	1256.93	< 0.0001	
A^2	3.09	1	3.09	162.27	< 0.0001	
B^2	2.21	1	2.21	115.97	< 0.0001	
**Residual**	0.5904	31	0.0190			
Lack of Fit	0.1188	4	0.0297	1.70	0.1791	not significant
Pure Error	0.4717	27	0.0175			
**Cor Total**	37.98	35				

Table S10. Total Phenolic Content model fit statistics

**Std. Dev.**	0.1380		**R^2**	0.9845
**Mean**	3.71		**Adjusted R^2**	0.9824
**C.V. %**	3.72		**Predicted R^2**	0.9763
			**Adeq Precision**	62.9434

Model for *Total Phenolic Content (mg GAE/g)* prediction in terms of actual factors:

$$TPC = -3.59 + 0.45(time) + 11.17(APD) - 0.02(time^2) - 5.50(APD^2)$$

Response Antioxidant Activity (FRAP method)

Table S11. Antioxidant Activity: FRAP ANOVA results

Source	Sum of Squares	df	Mean Square	F-value	p-value	
**Model**	22098.01	5	4419.60	249.36	< 0.0001	significant
A-time	4234.09	1	4234.09	238.90	< 0.0001	
B-APD	9779.33	1	9779.33	551.77	< 0.0001	
AB	1875.75	1	1875.75	105.83	< 0.0001	
A^2	2486.71	1	2486.71	140.30	< 0.0001	
B^2	4813.03	1	4813.03	271.56	< 0.0001	
**Residual**	531.71	30	17.72			
Lack of Fit	464.17	3	154.72	61.85	< 0.0001	significant
Pure Error	67.54	27	2.50			
**Cor Total**	22629.72	35				

Table S12. Antioxidant Activity: FRAP model fit statistics

**Std. Dev.**	4.21	**R^2**	0.9765
**Mean**	113.61	**Adjusted R^2**	0.9726
**C.V. %**	3.71	**Predicted R^2**	0.9628
		**Adeq Precision**	42.9031

Model for *Ferric Reducing Antioxidant Power (µmol FeSO4 eq/g)* prediction in terms of actual factors:

FRAP = – 167.35 + 18.40(time) + 515.15(APD) – 10.00(time.APD) – 0.46($time^2$) – 256.91(B^2)

Response Antioxidant Activity (DPPH method)

Table S13. Antioxidant Activity: DPPH ANOVA results

Source	Sum of Squares	df	Mean Square	F-value	p-value	
**Model**	2162.57	5	432.51	64.89	< 0.0001	significant
A-time	491.55	1	491.55	73.74	< 0.0001	
B-APD	1282.13	1	1282.13	192.35	< 0.0001	
AB	37.77	1	37.77	5.67	0.0238	
A^2	170.11	1	170.11	25.52	< 0.0001	
B^2	246.11	1	246.11	36.92	< 0.0001	
**Residual**	199.97	30	6.67			
Lack of Fit	192.59	3	64.20	234.87	< 0.0001	significant
Pure Error	7.38	27	0.2733			
**Cor Total**	2362.54	35				

Table S14. Antioxidant Activity: DPPH model fit statistics

**Std. Dev.**	2.58	**R^2**	0.9154
**Mean**	23.50	**Adjusted R^2**	0.9013
**C.V. %**	10.99	**Predicted R^2**	0.8658
		**Adeq Precision**	22.5694

Model for *DPPH Antioxidant Power (µmol TEAC/g)* prediction in terms of actual factors:

DPPH = – 46.15 + 4.25(time) + 119.10(APD) – 1.42(time.APD) – 0.12(time^2) – 58.09(APD^2)

Response Diluted Oleoresin Color Variation (ΔE)

Table S15. Diluted Oleoresin Color Variation ANOVA results

Source	Sum of Squares	df	Mean Square	F-value	p-value	
**Model**	1299.93	4	324.98	102.97	< 0.0001	significant
A-time	460.49	1	460.49	145.91	< 0.0001	
B-APD	721.91	1	721.91	228.74	< 0.0001	
A^2	35.22	1	35.22	11.16	0.0022	
B^2	100.70	1	100.70	31.91	< 0.0001	
**Residual**	97.84	31	3.16			
Lack of Fit	52.32	4	13.08	7.76	0.0003	significant
Pure Error	45.52	27	1.69			
**Cor Total**	1397.77	35				

Table S16. Diluted Oleoresin Color Variation model fit statistics

**Std. Dev.**	1.78		**R^2**	0.9300
**Mean**	13.11		**Adjusted R^2**	0.9210
**C.V. %**	13.55		**Predicted R^2**	0.9044
			**Adeq Precision**	29.9498

Model for *Oleoresin Color Variation (ΔE)* prediction in terms of actual factors:

$$\Delta E = -28.78 + 1.98(\text{time}) + 70.36(\text{APD}) - 0.05(\text{time}^2) - 37.16(\text{APD}^2)$$
